# Early stage epithelial ovarian cancer metastasis through peritoneal fluid circulation

**DOI:** 10.1186/s13048-021-00795-z

**Published:** 2021-03-16

**Authors:** Sigit Purbadi, Tricia Dewi Anggraeni, Angelina Vitria

**Affiliations:** Division of Gynecologic Oncology. Department of Obstetrics and Gynecology, Cipto Mangunkusumo Hospital, Jakarta, Indonesia

**Keywords:** Epithelial ovarian cancer, Proportion of spreading, Surgical staging

## Abstract

**Background:**

Although epithelial ovarian cancer (EOC) spreads through peritoneal circulation, all patients with clinical early-stage ovarian cancer (OC) benefit from routine surgical staging is still unclear.

**Methods:**

This cross-sectional study used data from medical records of patients with clinical early-stage EOC who received complete surgical staging from 2006 to 2016 at our hospital. We excluded patients with non-epithelial OC or with stage IV disease.

**Results:**

Among 50 patients with clinical early-stage EOC who underwent surgical staging, biopsies showed EOC cells in peritoneal fluid for 12 patients (24%), in peritoneal tissue for ten patients (20%), and omental tissue for eight patients (16%). Of those 50 patients, 40 patients had undergone peritoneal biopsies, and the other five patients also had omental biopsies. The results showed that only one (2.5%) from 40 patients with peritoneal biopsy and three (6.7%) from 45 patients with omental biopsy had no visible nodules. From cytology examination, 3 out of 26 patients (11.5%) showed positive cytology from peritoneal washing.

**Conclusions:**

Routine peritoneal biopsies do not seem advantageous for patients with clinical early-stage EOC as negative visible nodules with positive biopsy results were only 1 in 40 cases. However, further study with a larger cohort is needed to obtain more information on peritoneal fluid metastasis patterns.

## Introduction

Among gynecologic cancers, ovarian cancer (OC) causes major death in women. In 2018, an estimated 22,240 new OC diagnoses and 14,070 deaths from OC occurred in the United States [1–3]. About 70% of new OC cases are diagnosed at an advanced stage [3, 4], and 90% are epithelial ovarian cancer (EOC), whereas the remainder is non-epithelial [4–6]. About 50% of the OCs were diagnosed at an advanced stage. Advanced-stage OC has a worse prognosis than early-stage OC [4, 6].

Ovarian cancer commonly spreads to the abdominal cavity and forms implant tumors through peritoneal circulation [7]. These implant tumors are critical prognostic predictors as they indicate higher risks of recurrence and mortality compared with OC without implant tumors in the abdominal cavity. Implant tumors are also an essential determinant of whether additional therapy is required [8–10].

Therefore, definitive staging—i.e., surgical staging—is necessary for determining treatment for clinical early-stage OC. Surgical staging procedures include peritoneal washing, ovary removal, hysterectomy, lymphadenectomy, omentectomy, and peritoneal biopsy. The spread of OC through peritoneal fluid circulation is evaluated through cytological examination, peritoneal biopsy, and omentectomy [9–13].

Whether complete surgical staging, especially cytology, peritoneal biopsy, and omentectomy, should be routinely performed in all cases of clinical early-stage EOC (i.e., confined to the ovary only) is unclear. Thus, we conducted this study about OC spreading patterns through the peritoneal fluid circulation to evaluate whether surgical staging should be routinely performed for all patients with clinical early-stage EOC.

## Materials and methods

### Patients

This descriptive cross-sectional study used data from the medical records of patients with early-stage (tumor confined to the ovary) who underwent complete surgical staging at a single hospital from 2006 to 2016. We excluded patients with non-epithelial OC or stage IV disease. The mentioned complete surgical staging are cytology, peritoneal biopsy, and omentectomy including; abdominal and pelvic exploration, ascites findings, paraaortic lymphadenectomy, paracolic and diaphragm lymphadenectomy.

### Statistical methods

As no data on the prevalence of OC peritoneal spreading is available through previous studies, we used a cross-sectional study design with a categoric variable proportion set at 0.5. We set the false error rate at 15% (Zα 1.96). Medical records that met the inclusion criteria were collected and analyzed using SPSS 20 (IBM) software for Windows® operating systems. Histopathologic features, including differentiation, cytological results, peritoneal biopsy results, metastases to the omentum, and stage, are reported in percentages.

## Results

We collected medical record data from July 2016 to August 2017 from the hospital database. From 2006 to 2016, 2711 patients were diagnosed with EOC at this hospital, of whom 50 had clinical early-stage OC and underwent complete surgical staging for which peritoneal biopsy data were available. Peritoneal biopsy locations were right paracolic, left paracolic, Douglas cavity, pre-vesica, and diaphragm. Not all patients received diaphragm biopsies, and one patient received a peritoneal diaphragm biopsy.

From the data, we got the mean age of subjects was 46.7 ± 8.4 years. About half were multiparous, and 32% were nulliparous birth.

**Fig. 1:**
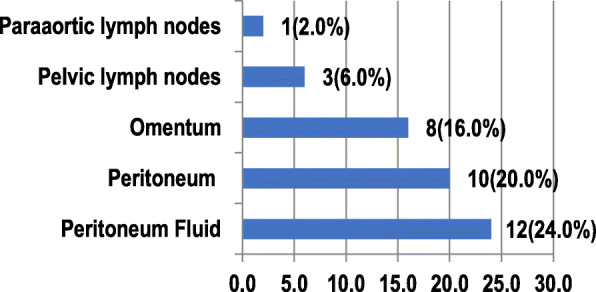
Percentages of ovarian cancer spreading in the peritoneum, omentum, peritoneal fluid, and lymph nodes (n = 50)

Among 50 patients with clinical early-stage EOC, surgical biopsies showed EOC cells in peritoneal fluid for 12 patients (24%), in peritoneal tissue for ten patients (20%), and omental tissue for eight patients (16%; Fig. 1). Three patients (6%) had cells in their pelvic lymph nodes, and one patient (2%) in her para-aortic lymph node (Fig. 1).

**Fig. 2:**
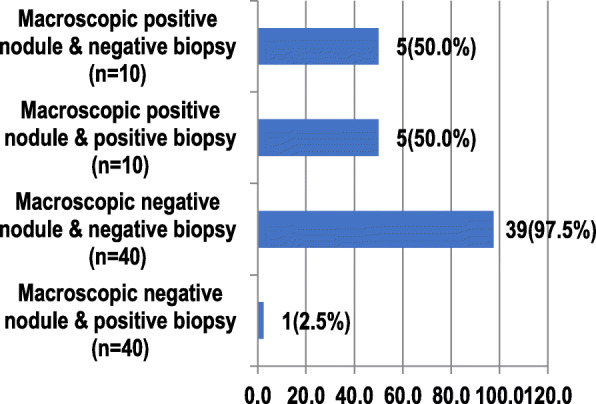
Comparison between intraoperative findings with peritoneal pathology anatomy findings (*n* = 50)

Among ten patients with macroscopic peritoneal nodules, half had positive biopsy results, and half had negative peritoneal biopsy results (50% and *n* = 5 for both; Fig. 2). We found one case (2.5%) of the visible negative nodule with positive biopsy results and 39 cases (97.5%) of the visible negative nodule with negative biopsy results.

**Fig. 3:**
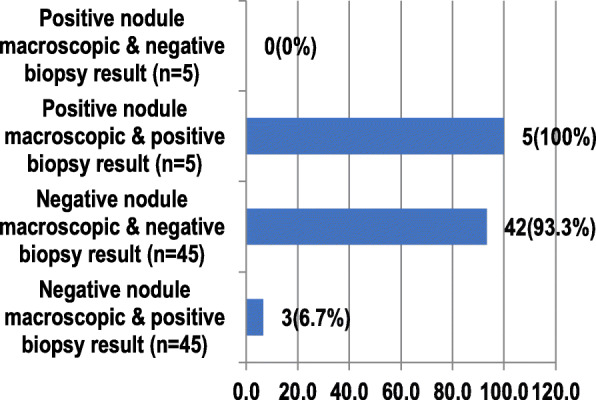
Comparison of intraoperative findings with omentum pathology anatomy findings (*n* = 50)

Referring to Fig. 3, this study shows five cases (100%) of the visible nodule in the omentum with positive biopsy results. From all visible nodules in the omentum, there were no cases (0%) of subjects with negative results, three cases (6.7%) of the visible negative nodule with positive biopsy results, and 42 cases (93.3%) of the visible negative nodule with negative results.

**Fig. 4:**
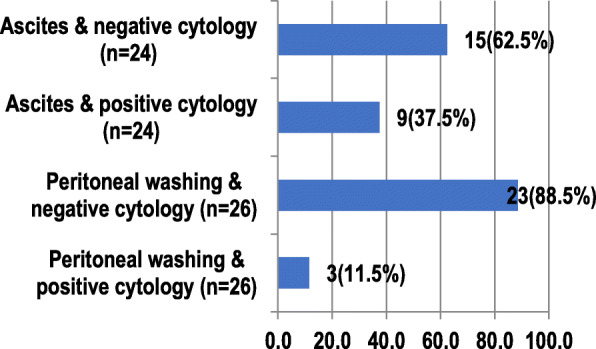
Comparison of intraoperative findings and cytology results (*n* = 50)

Among patients with ascites, nine (37.5%) had positive cytology results, and 15 (62.5%) had negative cytology results (Fig. 4). Among patients who underwent peritoneal washing, three (11.5%) had positive cytology results, and 23 (88.5%) had negative cytology results.

Based on the distribution of positive peritoneal biopsy results among patients, this study showed there were 3 of 7 cases of low-grade serous histopathology (Table 1). The high-grade serous and mucinous histopathology showed the same result (1 of 6 cases). The endometrioid histopathology resulted in 1 of 10 cases. There was no case on the clear cell. Positive results on peritoneal biopsy were also attained at all stage three cases where stage 3b resulted in 3 out of 3 cases, stage 3c was 2 of 4 cases, and stage 3a was 1 of 4 cases. For differentiation characteristics, this study showed 1st grade was 3 out of 20 cases, 2nd grade was 1 of 14 cases, and 3rd grade was 2 out of 16 cases.

**Table 1:** Staging, histopathology, and cancer differentiation findings by peritoneal biopsy, omentum biopsy, and cytology result in 50 subjects with ovarian cancer

Characteristics	Peritoneum (***n*** = 50)			Omentum			Fluid		
	Pos.	Neg.	Total	Pos.	Neg.	Total	Pos.	Neg.	Total
**Histopathology**									
High-grade serous	1	6	7	3	4	7	3	4	7
Endometrioid	1	10	11	2	9	11	3	8	11
Mucinous	1	6	7	0	7	7	0	7	7
Clear-cell	0	18	18	3	15	18	5	13	18
Low-grade serous	3	4	7	0	7	7	1	6	7
**Staging**									
Stage 1a	0	26	26	0	26	26	0	26	26
Stage 1b	0	2	2	0	2	2	0	2	2
Stage 1c	0	8	8	0	8	8	8	0	8
Stage 2a	0	3	3	0	3	3	0	3	3
Stage 2b	0	0	0	0	0	0	0	0	0
Stage 2c	0	0	0	0	0	0	0	0	0
Stage 3a	1	3	4	3	1	4	0	4	4
Stage 3b	3	0	3	1	2	3	0	3	3
Stage 3c	2	2	4	4	0	4	4	0	4
**Differentiation**									
Grade 1	3	17	20	4	16	20	2	18	20
Grade 2	1	13	14	3	11	14	4	10	14
Grade 3	2	14	16	1	15	16	6	10	16

Based on the distribution of positive omentum biopsy results among patients of this study, none had low-grade serous or mucinous histologic. Positive results on omentum biopsy were obtained at all stage three cases where stage 3a was 3 out of 4 cases, stage 3b was 1 in 3 cases, and stage 3c was 4 of 4 cases. There were 4 out of 20 cases of 1st grade in cell differentiation characteristics, 3 of 14 cases of 2nd grade, and 1 out of 16 cases of 3rd grade.

Based on the distribution of positive cytology cases, this study found no case in the mucinous histologic. Based on stage distribution, positive cytology was present in all cases of stage 3c and stage 1c. Based on cell differentiation characteristics, 1st grade was 2 out of 20 cases, 2nd grade was 4 out of 14 cases, and 3rd grade was 6 out of 16 cases (Table 1).

## Discussion

The most common and the earliest way of spreading EOC is through exfoliation along with the peritoneal surface. Cells spread directly to the peritoneum of the pelvis and abdomen. The spreading tends to follow the peritoneal fluid circulation path from the right paracolic direction toward the cephalad, i.e., the right hemidiaphragm attached to the peritoneum and omentum [9, 11, 13]. This study found that 24% (12/50) of patients had EOC cells in peritoneal fluid, 16% (8/50) in the omentum, and 20% (10/50) in the peritoneum.

Comparing intraoperative findings with peritoneal biopsy results, half of them had positive biopsy results from 10 patients with visible peritoneal nodules. Half had negative peritoneal biopsy results (50% and *n* = 5 for both). The proportion of visible negative nodules with a positive peritoneal biopsy result was one case (2.5%) of all subjects with visible negative nodules (40 cases); meanwhile, the proportion of visible negative nodules with a negative result was 39 cases (97.5%).

From the comparison between intraoperative findings with omentum biopsy results, all five patients (100%) with positive biopsy results also had positive visible nodules. In contrast, among those with visible negative nodules (*n* = 45), three (6.7%) had positive biopsy results, and 42 (93.3%) had negative results. These findings suggest that nodules in the peritoneum or omentum might not necessarily contain tumor cells. However, the presence of nodules in the peritoneum does indicate inflammation, which can cause cell proliferation.

Comparing intraoperative findings with ascites fluid or peritoneal washing cytology results, we found the proportion of ascites with positive cytology results was 9 cases (37.5%) of all subjects with ascites (24 cases). For subjects with ascites but have negative cytology results, we found 15 cases (62.5%). In comparison, the proportion of peritoneal washing with the positive result was 3 cases (11.5%) from all subjects who had peritoneal washing (26 cases). The proportion of peritoneal washing with negative cytology results were 23 cases (88.5%). These findings indicate that ascites fluid does not necessarily contain tumor cells, eventhough, ascites’ presence should be a particular concern for the operator that there could be a blockage in the abdominal cavity caused by inflammatory or cancerous cells.

The results of this study are in accordance with several existing studies where the rate of spread of stage III ovarian cancer through the lymphogen pathway reaches 42 to 78%. Whereas metastasis via the hematogenous route is rare in epithelial ovarian cancer and is often a late finding in this disease [9, 12]. A recent studies by Yousefi et al., stated that hematogenous metastasis still considered as a rare event in OC, eventhough the detection of hematogenic spreading (circulating tumor cells in blood) in early stages could give a valuable information such as impending metastases prediction and drug responses evaluation in EOC patients [14].

The distribution of peritoneal biopsy results showed that the most common positive results were low serous cancer, stage 3 disease, and grade 1 differentiation. The most common positive omentum biopsy results were high-grade serous histology, stage 3 disease, and grade 2 differentiation. The most common positive cytology results were high-grade serous cancer, stages 3c and 1c disease, and grade 3 differentiation. Our findings indicate that the most common EOCs to spread to the peritoneum, omentum, and peritoneal fluid were the high- and low-serous types. The spread of higher stage disease was commonly seen in the peritoneum and omentum, and differentiation grade did not seem to affect spreading patterns.

## Conclusion

Peritoneal biopsies do not appear to be useful for early-stage epithelial ovarian cancer due to low positive results in biopsies. Further research is needed with a larger sample to verify and expand on our results.

## Data Availability

The data used to support the findings of this study are included in the article.

## Abbreviations

EOC: Epithelial Ovarian Cancer
OC: Ovarian cancer
